# Household income and food addiction symptoms predict ultra-processed foods intake in Brazilian university students: implications for policy

**DOI:** 10.1186/s41155-026-00405-1

**Published:** 2026-07-01

**Authors:** Guilherme Macedo Soares Coutinho, Jasmin Bruna Stariolo, Thayane Carvalho Lemos, Camila Monteiro Fabricio Gama, Arthur Viana Machado, Daniela Silva Canella, Mirtes Garcia Pereira, Leticia de Oliveira, Isabel Antunes David

**Affiliations:** 1 Biomedical Institute, Universidade Federal Fluminense, Prof. Hernani Pires de Mello, 101 São Domingos, Niterói, RJ 24210-130 Brazil; 2 Department of Natural Sciences, Institute of Humanities and Health, Universidade Federal Fluminense, Rio das Ostras, RJ Brazil; 3 Department of Applied Nutrition, Institute of Nutrition, Universidade do Estado do Rio de Janeiro, Rio de Janeiro, Brazil

**Keywords:** Ultra-processed food, Income, Food addiction, Public health, Negative binomial regression

## Abstract

**Abstract:**

In Brazil, ultra-processed food consumption has increased disproportionately among lower-income groups, and food insecurity has been linked to food addiction. Previous studies have examined income and food addiction separately as predictors of ultra-processed food intake. This study evaluated the joint contribution of household income and food addiction to ultra-processed food intake in Brazilian university students.

**Methods:**

A cross-sectional online study was conducted with 368 Brazilian university students aged 18–30 years. Ultra-processed food intake was assessed using a score based on the Nova classification, reflecting the number of ultra-processed food subgroups consumed on the previous day. Food addiction was measured with the modified Yale Food Addiction Scale 2.0 and operationalized as diagnostic severity (none, mild, moderate, severe) and symptom presence (zero vs. one or more symptoms). Household income was categorized into four classes (A-D/E). Associations were examined using negative binomial regression, with multivariable models adjusted for body mass index and additional models including gender, race, and perceived stress related to the COVID-19 pandemic.

**Results:**

Lower household income was consistently associated with higher intake of ultra-processed foods. Compared with the highest income class, participants in income classes C and D/E showed expected intakes 39% and 42% higher, respectively. When food addiction was analyzed using diagnostic criteria, only the mild diagnosis group differed from the non-food addiction group, showing lower expected intake after adjustment for body mass index. In contrast, symptom-based analyses revealed that participants with one or more food addiction symptoms had an approximately 18% higher expected ultra-processed food intake than asymptomatic individuals. Lower income and symptom presence were particularly associated with consumption of ultra-processed foods that replace or accompany meals, as well as ultra-processed beverages.

**Conclusions:**

Household income and food addiction symptoms jointly influence ultra-processed food intake among Brazilian university students. Symptom-based measures appear more sensitive than diagnostic categories for identifying population-level vulnerability. These findings highlight the need for public policies that address both socioeconomic inequalities and addictive-like eating behaviors to reduce excessive ultra-processed food consumption.

**Supplementary Information:**

The online version contains supplementary material available at https://doi.org/10.1186/s41155-026-00405-1.

## Introduction

According to the Nova classification system, ultra-processed foods (UPF) are industrial formulations mainly composed of ingredients of exclusive industrial use and produced through multiple processing steps designed to maximize palatability and profitability (Monteiro et al., 2016, 2019). These products typically contain combinations of refined sugars, fats, sodium, and cosmetic additives that enhance sensory appeal and shelf life (Monteiro et al., 2016, 2019), and include packaged snacks, soft drinks, fast foods, and ready-to-eat meals (Pan American Health Organization [PAHO], 2019). UPF are aggressively marketed and widely distributed, contributing to their growing dominance in global food systems (Monteiro et al., 2013).

A substantial body of evidence links higher UPF consumption to increased risk of noncommunicable diseases, mental health problems, and all-cause mortality (Hall et al., 2019; Lane et al., 2024; Pagliai et al., 2021). Beyond health outcomes, UPF-centered diets are also associated with environmental harms, including increased greenhouse gas emissions, packaging waste, and biodiversity loss (Anastasiou et al., 2022). Together, these impacts position UPF consumption as a central driver of the syndemic of obesity, undernutrition, and climate change (Swinburn et al., 2019), reinforcing the need to better understand the determinants of consumer food choices within contemporary food environments.

In Brazil, although absolute UPF consumption remains higher among individuals with greater income and education, national data indicate a relatively small increase over the last decade, from 18.7% to 19.7% of total energy intake between 2008 and 2009 and 2017–2018; notably, the most significant increases occurred among lower-income and socially vulnerable groups, whereas consumption declined among the highest income quintile (Louzada et al., 2023). This pattern, observed in national dietary surveillance data, has been partly attributed to structural factors discussed in the literature, including declining UPF prices, persistent income inequality, and reduced access to unprocessed or minimally processed foods among lower-income populations (Machado et al., 2017; Maia et al., 2020). These disparities were exacerbated during the COVID-19 pandemic, when income loss, food insecurity, and rising prices of staple foods disproportionately affected lower-income households, contributing to greater reliance on UPF (Andrade et al., 2023; Rede Brasileira de Pesquisa em Soberania e Segurança Alimentar e Nutricional, 2021; Silva-Júnior et al., 2022).

In parallel, growing evidence suggests that UPF may elicit addictive-like eating behaviors due to their unique combination of highly rewarding ingredients and rapid absorption profiles (Gearhardt & Schulte, 2021; Lemos et al., 2026; Schulte et al., 2015). Although food addiction is not formally recognized as a diagnostic entity in the DSM-5, evidence indicates that excessive UPF consumption is accompanied by behavioral patterns analogous to substance-use-related criteria, such as loss of control, craving, tolerance, and continued use despite negative consequences (Gearhardt et al., 2016; Gearhardt & DiFeliceantonio, 2023). These behaviors are commonly assessed using the Yale Food Addiction Scale (YFAS; Gearhardt et al., 2009) or its shorter version, the modified YFAS (mYFAS; Schulte & Gearhardt, 2017), which has been validated for use in Brazil (Nunes-Neto et al., 2018). However, few studies have jointly examined food addiction symptoms and UPF intake using both YFAS-based measures and the Nova framework (Filgueiras et al., 2019; Silva-Júnior et al., 2023; Whatnall et al., 2022).

Significantly, food addiction symptoms and socioeconomic disadvantage may interact. Food insecurity and psychological stress have been associated with higher vulnerability to addictive-like eating behaviors, particularly during the COVID-19 pandemic (Hong et al., 2020; Leung et al., 2022; Silva-Júnior et al., 2022). While household income and food addiction symptoms have each been linked independently to UPF consumption, their joint contribution remains poorly understood, especially in socially heterogeneous populations such as Brazil. Taste and price are key determinants of food choice (Darmon et al., 2003; Johnson & Wardle, 2014). UPF are typically engineered to maximize sensory appeal through combinations of fats, sugars, salt, and additives while remaining relatively inexpensive, which may enhance incentive-motivational responses similar to those observed in individuals with substance use disorders (Dagher, 2009; Fazzino et al., 2023; Gupta et al., 2019). These characteristics suggest that economic constraints and quasi-addictive motivational processes may jointly shape UPF consumption.

Higher household income may not automatically translate into a healthier diet. However, it has been argued that diet quality can be better explained when other compelling factors that compound economic restraints (such as psychological stress) are accounted for alongside household income (Laraia et al., 2017). Therefore, the present study aimed to investigate the individual and combined effects of household income and food addiction symptoms on UPF consumption among Brazilian university students, leveraging the socioeconomic and racial heterogeneity characteristic of the Brazilian population (Salata, 2020). By examining these factors concurrently, this study seeks to clarify how socioeconomic and psychological vulnerabilities intersect to shape UPF intake, with implications for public health policies aimed at reducing dietary inequalities and excessive consumption of ultra-processed foods.

## Methods

### Participants and study design

The participants were undergraduate students from the Brazilian public university Universidade Federal Fluminense, located in the Southeast region of Brazil. According to the Center for World University Rankings, Brazil’s public universities are the country’s best academic performers (Center for World University Rankings, 2023). As they are free and of good quality, individuals from various income brackets compete for places at these universities. Among Brazil’s public universities, Federal Universities, such as Universidade Federal Fluminense, have the most comprehensive diversity and inclusion policies, which favor the entry and retention of undergraduate students from diverse income levels and races (Silva, 2019).

Universidade Federal Fluminense is one of Brazil’s Federal Universities with the largest number of undergraduate students (Instituto Nacional de Estudos e Pesquisas Educacionais Anísio Teixeira, 2022). Using a sample from a federal university enabled us to control for sociodemographic factors that could bias the interpretation of income results. For example, the individuals in the sample had the same level of education and lived in the same region of Brazil (the Southeast), but had varying incomes. We conducted a web-based cross-sectional study during a later phase of the COVID-19 pandemic, from May to November 2021, when virtual classes were still in the instructional format. A convenience sampling approach was applied in this study. Students were invited through a recruitment campaign on Instagram, WhatsApp, and Facebook, and received access via email to a Google Form link where the data were gathered. The participants were instructed to complete a set of questionnaires composed of sociodemographic, gender, anthropometric, and dietary pattern questions and psychometric scales. To calculate the sample size, 95% confidence intervals and a 5% acceptable margin of error were adopted for a population of 40.000 (the approximate number of undergraduate students at Universidade Federal Fluminense). The sample size estimated was 381 participants.

The inclusion criteria included being between 18 and 30 years of age and being a native Portuguese speaker. The exclusion criteria included reporting having a diagnosis of an eating disorder and/or being pregnant. Compared to nonclinical individuals, patients with eating disorders have a far greater frequency of food addiction (Wiss, 2022); thus, we concentrated the analysis on a nonclinical sample. In addition, dietary behavior changes during pregnancy (Iordachescu et al., 2020); thus, we also excluded pregnant participants.

Ethical approval for the involvement of human subjects in this study was granted by the Ethics Research Committee. Participants gave informed consent before taking part in the study.

### Assessment instruments

#### Nova-UPF score: assessment of UPF consumption

The Nova-UPF score (Costa et al., 2021) is based on the Nova classification system, a descriptive criterion for classifying food according to the nature, extent, and purpose of industrial processing (Monteiro et al., 2016). It offers an inexpensive, easy, fast method to assess the consumption of UPF and is a good option for tracking UPF consumption in low- and middle-income countries (Correa-Madrid et al., 2023; Costa et al., 2021) and in Brazilian health surveys (Costa et al., 2022). The scale contains twenty-three subgroups, which are grouped into three main categories: products that replace or go with meals (ten subgroups; e.g., sausage, hamburger, or chicken nuggets); beverages (six subgroups; e.g., traditional or diet soft drinks); and snacks (seven subgroups; e.g., branded ice cream or popsicle). These categories include UPF, which highly contribute to daily energy intake, as estimated by the Household Budget Survey (Pesquisa de Orçamentos Familiares, POF after its Portuguese acronym), which was conducted by the Brazilian Institute of Geography and Statistics between 2008 and 2009 (IBGE, 2011). Participants were asked to select the items they had consumed the previous day. The final score consisted of the sum of all the items selected in the three categories, ranging from 0 to 23. See Costa et al. (2021) for the three categories and twenty-three subgroups of the Nova-UPF score. Recently, the Nova-UPF score has been applied in the NutriNet-Brasil, a very relevant ongoing web-based cohort study in Brazil (Santos et al., 2023).

#### Anthropometric, demographic, and household income data

Anthropometric data consisted of reported weight (kilograms) and height (meters). Body mass index (BMI) was determined using the standard equation (weight [kg] / height*²* [m*²*]). The sample was stratified into groups of individuals who were underweight, normal weight, overweight, and obese, according to World Health Organization cutoffs (World Health Organization, 2000).

The demographic questionnaire included items such as gender (male, female, nonbinary, or other), race (black, white, yellow, indigenous, or nondeclared), and major area of study (e.g., from human science, biomedical science, or hard science).

One variable of interest in this study was household income. According to the Brazilian Institute of Geography and Statistics, *“The household is defined as a person living alone*,* or a group of persons connected by blood*,* domestic dependence or cohabitation norms living in a private housing unit”* (IBGE, 2022). Therefore, in this context, “household income” denotes both a student’s income and their private housing unit. The household income classes were based on the minimum wage in Brazil, calculated on a monthly basis (less than 2, 2–5, 5–10, 10 or more minimum wages per month). In 2021, when the data were collected, the minimum wage in Brazil was 1,100 Brazilian reais per month (approximately 220 US dollars at the August 2021 exchange rate, when one US dollar was equivalent to approximately 5 Brazilian reais). We divided the sample into four income groups: (1) participants from Class A came from families earning more than 10 minimum wages per month, and (2) participants from Class B came from families earning the equivalent of 5–10 minimum wages per month. (3) Participants from Class C came from families earning the equivalent of 2–5 minimum wages per month. (4) Participants from Class D/E came from families earning less than 2 minimum wages per month. This division was adapted from the Brazilian Institute of Geography and Statistics classification, which groups social classes into 5 categories based on monthly family income. Additionally, studies based on information from the National Household Sample Survey (Pesquisa Nacional por Amostra de Domicílios, PNAD, after its Portuguese acronym) suggest that at higher income levels (above 10 minimum wages), food insecurity becomes negligible (e.g., Hoffmann, 2008). Therefore, individuals with a household income above 10 minimum wages were considered Class A, and the other classes were adjusted accordingly.

#### Modified yale food addiction scale 2.0 (mYFAS 2.0)

The Modified Yale Food Addiction Scale 2.0 (mYFAS 2.0; Schulte & Gearhardt, 2017) is a brief version of the YFAS 2.0 (Gearhardt et al., 2016). Nunes-Neto et al. (2018) adapted and validated the mYFAS 2.0 instrument for use in Brazil. The mYFAS 2.0 contains 13 items. The score options are 0 (never), 1 (less than monthly), 2 (once a month), 3 (2–3 times a month), 4 (once a week), 5 (2–3 times a week), 6 (4–6 times a week), and 7 (every day). The goal of the scale is to evaluate eating behaviors that resemble addiction based on the Diagnostic and Statistical Manual of Mental Disorders, fifth edition (DSM-5; American Psychiatric Association, 2013). An example of an item from the mYFAS 2.0 is: “I tried and failed to cut down on or stop eating certain foods.”

Although the Portuguese wording of the mYFAS 2.0 validated by Nunes-Neto et al. (2018) was used, the scoring procedure adopted in the present study strictly followed the original frequency-based threshold criteria proposed by Schulte and Gearhardt (2017). This approach was chosen instead of relying on the numerical scoring table of the Brazilian adaptation to address concerns raised in a comment suggesting that numeric thresholds may underestimate symptom endorsement (Silva-Júnior et al., 2023) To ensure psychometric adequacy, internal consistency of the mYFAS 2.0 symptom scores was assessed, indicating excellent reliability in the present sample (Cronbach’s α = 0.88).

The modified Yale Food Addiction Scale 2.0 (mYFAS 2.0) provides two complementary outcomes: (1) a symptom count score ranging from 0 to 11 symptoms, reflecting the number of addiction-like eating behaviors endorsed; and (2) a categorical classification of food addiction severity (mild, moderate, or severe), which requires the presence of clinical significance in addition to symptom thresholds (Schulte & Gearhardt, 2017). In this study, both outcomes were examined to provide a comprehensive assessment of food addiction-related behaviors. Diagnostic criteria were included to ensure comparability with the existing literature and to anchor the construct within its validated clinical framework. However, emphasis was also placed on the symptom count score because it captures the full spectrum of problematic eating behaviors, including subclinical manifestations that may already be associated with increased vulnerability to UPF consumption. This perspective aligns with emerging evidence that addictive-like responses to UPF have important implications for population-level health risk and prevention, even in the absence of a formal diagnosis (Gearhardt et al., 2023), and is consistent with the Research Domain Criteria (RDoC) framework, which emphasizes continuous variation from normative to pathological functioning (Insel et al., 2010).

In population-based samples, YFAS-based symptom counts typically show a highly right-skewed distribution with a substantial mass at zero, as most individuals report no addiction-like symptoms (e.g.,Hauck et al., 2017). Inspection of the distribution in the present sample confirmed these characteristics, limiting the suitability of continuous modeling due to violated distributional assumptions and the risk of unstable parameter estimates. Therefore, symptom counts were dichotomized into asymptomatic (0 symptoms) and symptomatic (≥ 1 symptom) groups to improve model fit and interpretability in regression analyses (Green, 2021). From a public health perspective, this categorization is meaningful because the presence of even a single addiction-like symptom may signify vulnerability to maladaptive eating behaviors that may contribute to unhealthy dietary patterns at the population level.

#### Coronavirus stress measure scale

The Perceived Stress Scale (Cohen et al., 1983) was translated and verified in Portuguese by Luft et al. (2007) and is the basis for the Coronavirus Stress Measure (CSM) scale (Arslan et al., 2021). To assess the impact of the COVID-19 pandemic on people’s perceived stress, eight items are included in the scale that indicate the frequency with which an individual has felt a particular way owing to the COVID-19 pandemic (e.g., “How often have you been upset because of the COVID-19 outbreak?”). The scale has five points, with 0 representing never and 4 representing always. The total score ranges from 0 to 20. The greater the overall score is, the greater the perceived stress promoted by the COVID-19 pandemic is. This scale was used to evaluate the relationship between individuals’ perceived stress due to the COVID-19 pandemic and their symptoms of addiction to UPF. The Cronbach’s α in the present study was = .86.

### Data analysis

All the statistical analyses were performed using Jamovi (version 2.3; Jamovi Project, 2023), and the level of statistical significance was set at *p* < .050.

#### Sample characteristic

The demographic and anthropometric characteristics and food addiction symptoms of the sample were summarized using descriptive statistics. Continuous variables were displayed as medians and interquartile ranges, and categorical variables were displayed as frequencies.

Race (skin color) was self-reported according to the official categories adopted by the Brazilian Institute of Geography and Statistics (IBGE): ‘Branca’ (White), ‘Preta’ (Black), ‘Parda’ (Brown), ‘Amarela’ (Yellow/Asian), ‘Indígena’ (Indigenous), or non-declared. In the Brazilian context, individuals commonly referred to as “mixed race” in international literature are captured within the Brown category based on self-identification. For analytical purposes, participants who self-identified as Black or Brown were grouped into a single category labeled “Black”. This approach is widely used in Brazilian demographic, socioeconomic, and public health research and is consistent with national census data, which indicate that 55.5% of the Brazilian population identifies as Black (Black plus Brown) (IBGE, 2023).

In addition, the distribution of food addiction diagnostic severity (mild, moderate, severe) was descriptively examined across body mass index (BMI) categories. This approach was adopted in light of emerging evidence that the relationship between food addiction and BMI is non-linear and behaviorally heterogeneous, with meaningful variability across weight classes, particularly at lower severity levels (Wiss, 2022). Food addiction severity levels were cross tabulated with BMI categories, and relative frequencies were calculated to characterize how diagnostic severity varied across the BMI spectrum. These data were summarized visually using stacked bar charts.

Previous studies have shown that, in Brazil, the mean income of Black individuals is significantly lower than that of White individuals (Salata, 2020). Thus, we separated the household income frequency data by White and Black individuals and applied a chi-square test to investigate the distribution of household income by race in our data.

#### Negative binomial regression

The Shapiro‒Wilk test indicated that the Nova-UPF score did not follow a normal distribution (*W* = 0.95; *p* < .001). The Nova-UPF score variable is a count (i.e., nonnegative whole numbers) since it reflects the number of UPF consumed on the previous day. Thus, we opted to use a negative binomial regression, as it is considered a good fit for overdispersed count data (Green, 2021). Model fit was further supported by the Akaike Information Criterion (*AIC*; Akaike, 1998), which was lower for the negative binomial models than for the Poisson models for both the symptom-count (*AIC* = 1535.97 vs. 1559.61) and diagnostic-criteria analyses (*AIC* = 1542.16 vs. 1566.66), indicating superior fit. The incidence rate ratio (*IRR*), which is the percentage increase or decrease in the dependent variable per unit change in the independent variable, is obtained from the exponentiated regression coefficients in the negative binomial regression model.

First, we performed bivariate negative binomial regressions to evaluate the association between the independent variables of interest (food addiction based on diagnostic criteria or symptom count; household income) and possible confounders (gender, race, BMI, CSM scores) and the dependent variable (Nova-UPF score). For gender, we included only men and women in the analysis, as fluid and nonbinary individuals accounted for only 1.6% of the sample. For race, we included only Black and White individuals because Yellow and nondeclared individuals accounted for only 1.9% of the sample, and there were no data on Indigenous individuals. Variables with *p* values less than 0.200 and confidence intervals that did not present a null value (i.e., confidence intervals did not include 1) were included in the multivariable model.

Food addiction was examined using two complementary operationalizations, derived from the same instrument, to address distinct analytical aims: (i) a categorical classification based on diagnostic criteria (food addiction severity: non-food addiction, mild, moderate, and severe food addiction individuals) and (ii) symptom presence based on symptom count. Separate multivariable negative binomial models were estimated for each operationalization, following identical modeling procedures.

##### Diagnostic criteria: food addiction severity

In a first set of models, food addiction was operationalized according to diagnostic criteria, classified as non-food addiction, mild, moderate, or severe. The independent variables of interest were food addiction severity and household income (Classes A, B, C, and D/E), and the dependent variable was the Nova-UPF score (range: 0–23). The prevalence of food addiction diagnosis was reported in the descriptive statistics (Table 1).

**Table 1:** Characteristics of the whole sample (*n* = 368)

	*n* (%)
Food addiction diagnosis*	
Mild	22 (6.0)
Moderate	16 (4.3)
Severe	16 (4.3)
Food addiction symptoms’ count*	
Symptomatic (one or more symptoms)	175 (47.6)
Asymptomatic (zero symptoms)	193 (52.4)
Household income (class)**	
A	40 (10.9)
B	81 (22.0)
C	125 (33.9)
D/E	122 (33.2)
Gender	
Male	115 (31.3)
Female	247 (67.1)
Nonbinary or other	6 (1.6)
Race	
Black	142 (38.5)
White	219 (59.5)
Yellow	3 (0.8)
Nondeclared	4 (1.0)
Indigenous	0 (0.0)
Major area of study	
Human Science	161 (43.8)
Biomedical Science	125 (34.0)
Hard Science/ Engineering	82 (22.2)
BMI***	
Underweight (< 18.5 kg/m^2^)	42 (11.4)
Eutrophic (18.5–24.9 kg/m^2^)	228 (61.9)
Overweight (25–29.9 kg/m^2^)	64 (17.4)
Obesity (≥ 30 kg/m^2^)	34 (7.8)

*The Food Addiction diagnosis was based on the diagnostic criteria from the mYFAS 2.0. The Food Addiction symptom count was based on the number of items selected on the 11 nondiagnostic questions of the mYFAS 2.0 (symptom count score). The continuous data were dichotomized into two groups: asymptomatic (zero symptoms) or symptomatic (one or more symptoms)

** Class A: participants from families earning the equivalent of 10 minimum wages per month; Class B: participants from families earning the equivalent of 5–10 minimum wages per month; Class C: participants from families earning the equivalent of 2–5 minimum wages per month; and Class D/E: participants from families earning less than 2 minimum wages per month. The minimum wage in Brazil is 1,100 Brazilian reals per month (approximately 220 US dollars)

***Body mass index (BMI) cutoff was based on the World Health Organization (2000)

Multivariable models evaluated the joint association of food addiction severity and household income with UPF consumption. BMI was included as a covariate because it was associated with the Nova-UPF score in bivariate analyses (*IRR* = 1.02, 95% CI [1.00, 1.03], *p* = .037). Associations were tested in both crude and BMI-adjusted models. Results are presented as *IRR*s with 95% confidence intervals. Food addiction severity and household income were associated (χ² (9) = 21.0, *p* = .013). No evidence of substantial collinearity was observed between food addiction severity and household income.

##### Food addiction symptom count

Food addiction symptom counts were operationalized as a dichotomous variable (asymptomatic: 0 symptoms; symptomatic: ≥1 symptom) due to the highly skewed and zero-inflated nature of the symptom distribution (Green, 2021). Negative binomial regression models examined the association between symptom presence (e.g., asymptomatic: 0 symptoms; symptomatic: ≥ 1 symptom) and household income with the Nova-UPF score (range: 0–23). Multivariable models followed the same structure as those used for diagnostic criteria, including BMI as a covariate. Both crude and BMI-adjusted models were estimated, and results are expressed as *IRR*s with 95% confidence intervals. No significant association was observed between the presence of food addiction symptoms and household income (χ² (3) = 0.33, *p* = .950).

#### Differences in perceived stress between symptomatic and asymptomatic individuals (based on symptom count scores)

Psychological stress during the COVID-19 pandemic may have fueled food addiction symptoms in Brazilian undergraduate students (Silva-Júnior et al., 2022). Since the data were collected during the COVID-19 pandemic, we conducted an analysis to investigate whether individuals with one or more symptoms of food addiction would also present greater scores on the Coronavirus Stress Measure scale. We performed the Mann‒Whitney *U* test to compare the CSM scores between the symptomatic sample (one or more symptoms of food addiction) and the asymptomatic sample (zero symptoms of food addiction). This analysis focused on symptom count rather than diagnostic classification, as we aimed to examine whether psychological stress is associated with the presence of early or subthreshold symptoms rather than with categorical diagnostic status.

#### Race, gender, and stress

Race (*IRR* = 1.06, 95% CI [0.92, 1.22], *p* = .430), gender (*IRR* = 1.05, 95% CI [0.91, 1.22], *p* = .510) and CSM score (*IRR* = 1.01, 95% CI [0.99, 1.01], *p* = .480) were not included in the multivariable negative binomial regression because they were not related to the dependent variable in this sample. However, to further understand the possible influence of these variables on UPF intake, we included an additional multivariable negative binomial regression analysis adjusted for all the abovementioned variables in the supplemental material (supplemental Tables 1 and 2).

#### Frequency of UPF categories consumed the previous day according to food addiction symptoms count and household income

Associations between UPF categories consumed on the previous day and food addiction symptom presence (symptomatic vs. asymptomatic) and household income (Classes A, B, C, D/E) were examined using chi-square tests. UPF categories were defined according to the Nova-UPF score and included products that replace or accompany meals, beverages, and snacks. Participants were classified as having consumed a given category (“Yes”) if they reported intake of one or more subgroups within that category on the previous day; otherwise, they were classified as “No”. This approach allowed us to assess whether variations in UPF consumption across income and food addiction symptoms differed by UPF category.

Category-level analyses were restricted to the symptom count operationalization of food addiction, which provides greater sensitivity to subthreshold variability. Diagnostic criteria were examined in the main regression models and were not reanalyzed at the category level to avoid redundancy.

## Results

### Sample characteristics

**Fig. 1:**
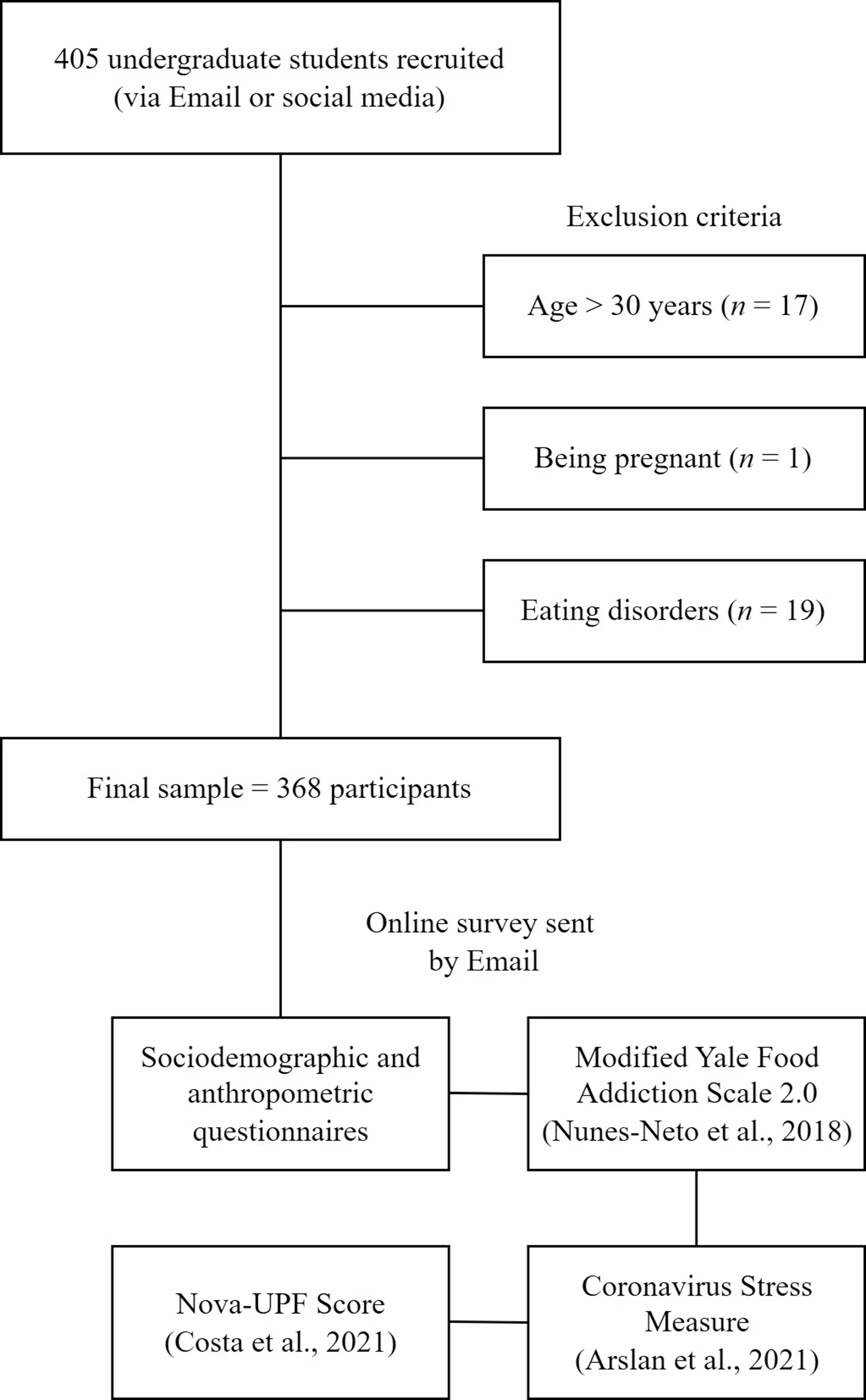
Flowchart of Participant Recruitment, Exclusion Criteria, and Study Procedures*.* A total of 405 undergraduate students were recruited via email and social media. After exclusion based on age (> 30 years), pregnancy, and self-reported eating disorders, the final sample consisted of 368 participants who completed online questionnaires assessing sociodemographic and anthropometric characteristics, food addiction symptoms, ultra-processed food consumption, and COVID-19-related psychological stress

A sample of 405 participants participated in the cross-sectional study. A total of thirty-seven individuals were eliminated due to their age (*n* = 17), reporting an eating disorder diagnosis (*n* = 19), and/or pregnancy records (*n* = 1). Thus, the final study sample included 368 participants (247 women; *M*_age_ = 21.8; *SD* = 2.9). The sample was described in a previous study; more information on the exclusion criteria and questionnaires used may be found in the study by Stariolo et al. (2024) and in Fig. 1.

The sample characteristics are summarized in Table 1. The participants (*M*_age_ = 21.8, *SD* = 2.9; *n* = 303) were primarily female (female = 67.1%; men = 31.3%; nonbinary or other = 1.6%) and white (*n* = 219; 59.5%) and included more individuals from Classes D/E and C (33.2% and 33.9%, respectively).

The reported mean body mass index (BMI) of the participants was 23.1 kg/m² (*SD* = 4.6 kg/m²; BMI_min_ = 14.8 kg/m²; BMI_max_ = 43.1 kg/m²). Most of the participants were of normal weight (*n* = 228, 61.9%). A total of 54 (14.7%) participants met the criteria for food addiction, 22 (6%; 19 women) were classified as mild, and 16 (4.3%; 11 and 14 women) were classified as moderate and severe. These results are consistent with those reported by studies of Brazilian university samples, in which a higher percentage of female, white, higher-income, and normal weight individuals were also observed (e.g., Guedes & Silva, 2021). To better determine if the sample in the present study represented the research setting population (the total number of students at the University where the sample was recruited), we included a table in the supplementary material with population information (supplemental Table 3), as recommended by Sousa et al. (2004).

**Fig. 2:**
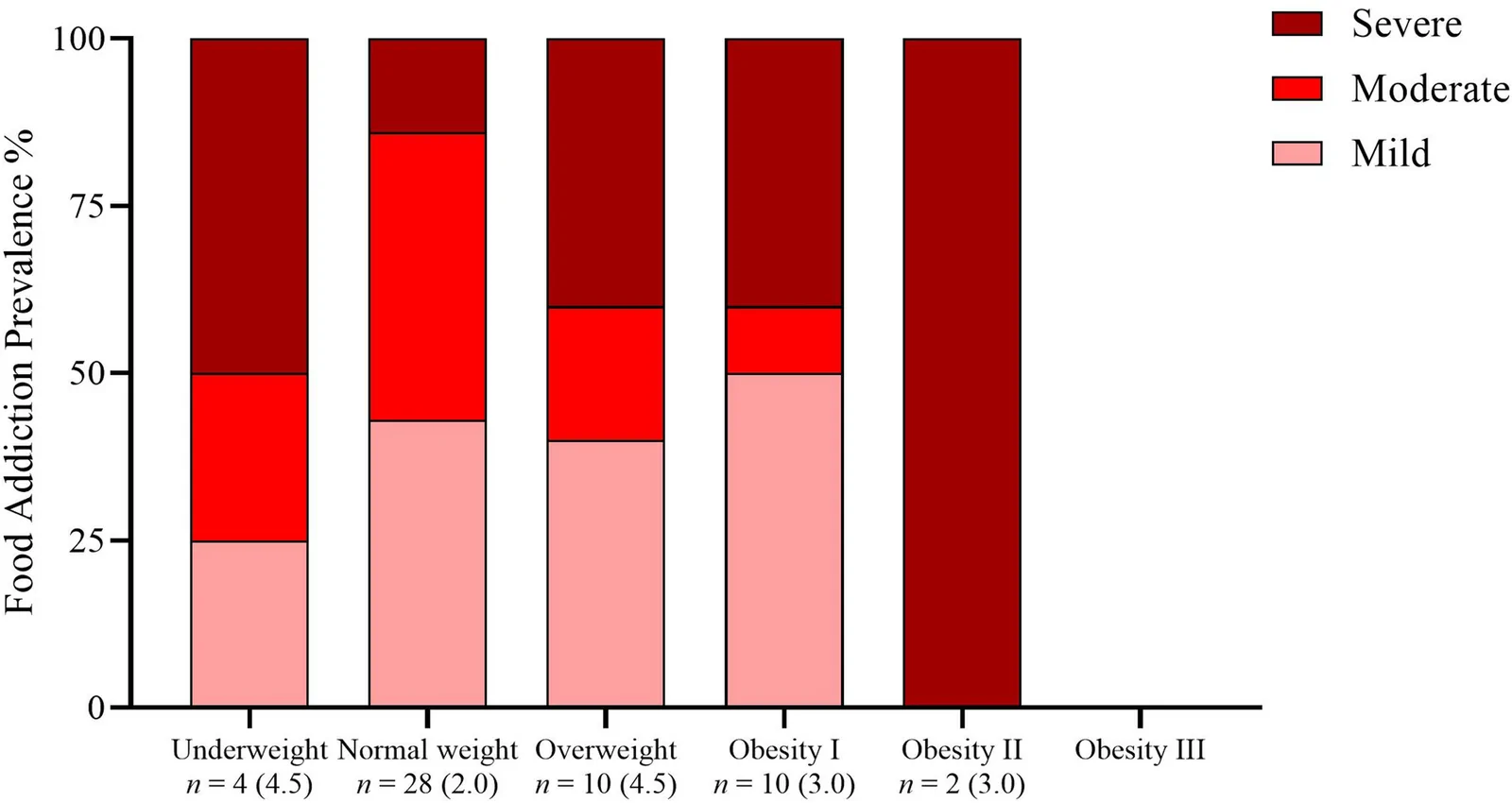
Distribution of Food Addiction Diagnostic Severity Across Body Mass Index (BMI) Categories (*n* = 368). Mild food addiction was observed across normal weight, overweight, and obesity class I categories, whereas severe food addiction was predominantly concentrated in higher obesity classes, illustrating heterogeneity in diagnostic severity across BMI levels

As shown in Fig. 2, mild food addiction was distributed across normal weight, overweight, and obesity class I categories. In contrast, severe food addiction was predominantly observed among individuals with higher BMI, particularly obesity class II. Moderate food addiction was less frequent and showed no clear concentration within a single BMI category.

**Fig. 3:**
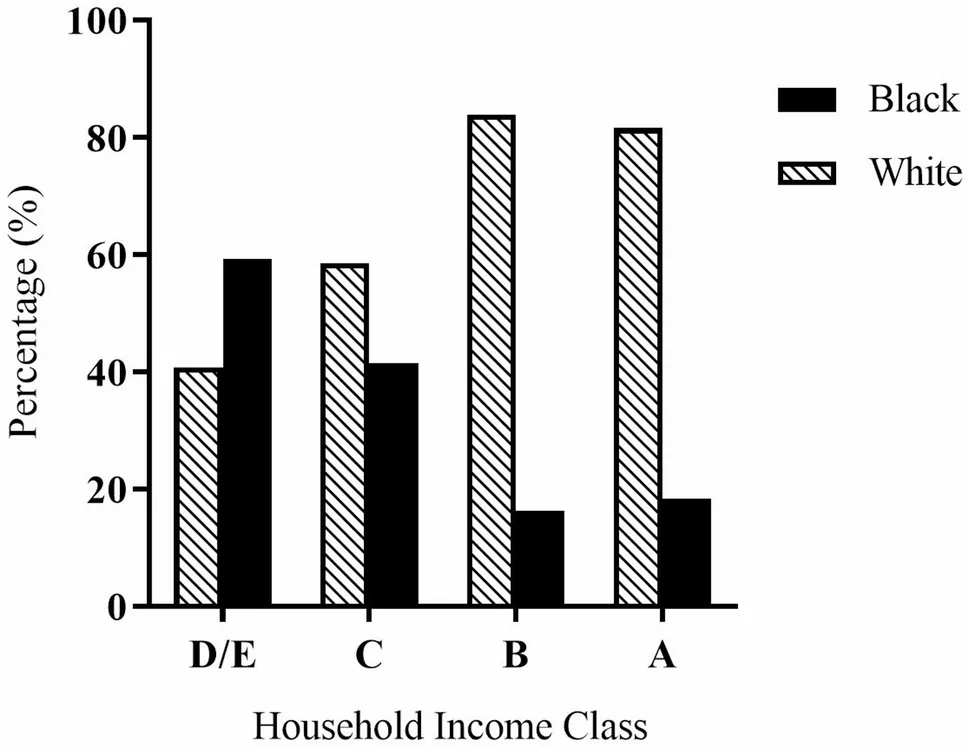
Race distribution (%) across household income classes (*n* = 361). Household income was categorized as Classes A, B, C, and D/E. The Black category includes individuals self-identified as Black or Brown according to Brazilian census classifications

As shown in Fig. 3, the chi-square test revealed that there was an association between race (White or Black) and household income (Classes A, B, C, D/E; χ² (3) = 44.8; *p* < .001). There was a greater percentage of Black individuals than White individuals in Class D/E (59.2% vs. 40.8%), while the inverse pattern was observed in Class A (18.4% of Black individuals vs. 81.6% of White individuals). Yellow, Indigenous, and nondeclared represented only 1.8% (*n* = 7) of the sample. Thus, the analyses included 361 participants.

### Negative binomial regression

Yellow, Indigenous and nondeclared (race), as well as nonbinary and others (gender) represented only 3.4% (*n* = 13) of the sample (Table 1), and were excluded from the analyses. Thus, the negative binomial regression analyses included 355 participants.

#### Household income and food addiction severity (based on diagnostic criteria)

**Fig. 4:**
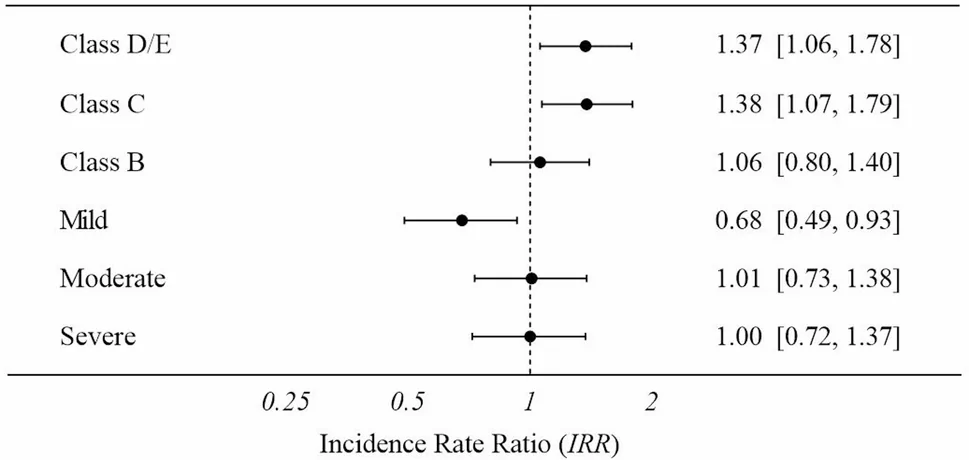
Forest plot of adjusted incidence rate ratios (IRR) for ultra-processed food intake based on food addiction diagnostic severity and household income (*n* = 355). The Nova-UPF score was the dependent variable in negative binomial regression models. Reference categories were no food addiction and income Class A. Points represent *IRR*s and horizontal lines indicate 95% confidence intervals

As shown in Table 2, household income was consistently associated with UPF consumption, with participants in income classes C and D/E exhibiting higher expected UPF consumption than those in class A across all models. Regarding food addiction diagnosis, only the mild diagnosis group differed significantly from the non-food addiction reference group, showing a lower expected number of UPF subgroups consumed on the previous day in the BMI-adjusted model (*IRR* = 0.68, 95% CI [0.49, 0.93], *p* = .019). Figure 4 illustrates the adjusted incidence rate ratios and corresponding confidence intervals, highlighting positive associations for lower income classes and an inverse association for the mild food addiction diagnosis. In contrast, the moderate and severe diagnosis groups were not significantly associated with UPF consumption, likely reflecting limited statistical power due to small group sizes.

**Table 2:** The impact of income (Classes A, B, C, D/E) and food addiction diagnosis (Mild, Moderate or Severe) on UPF consumption: bivariate and multivariable negative binomial regression models

UPF consumption	Bivariate Model			Multivariable Model (Crude)			Multivariable Model (adjusted for BMI)		
	IRR	95% CI	*p* value	IRR	95% CI	*p* value	IRR	95% CI	*p* value
Income									
Class D/E	1.40	[1.09, 1.82]	**0.010**	1.37	[1.06, 1.78]	**0.016**	1.37	[1.06, 1.78]	**0.015**
Class C	1.44	[1.12, 1.87]	**0.005**	1.40	[1.09, 1.82]	**0.010**	1.38	[1.07, 1.79]	**0.014**
Class B	1.09	[0.83, 1.45]	0.526	1.06	[0.80, 1.40]	0.701	1.06	[0.80, 1.40]	0.692
Food Addiction									
Mild	0.71	[0.51, 0.98]	**0.039**	0.71	[0.51, 0.97]	**0.036**	0.68	[0.49, 0.93]	**0.019**
Moderate	1.07	[0.77, 1.48]	0.664	1.01	[0.73, 1.39]	0.932	1.01	[0.73, 1.38]	0.949
Severe	1.11	[0.80, 1.52]	0.523	1.06	[0.77, 1.44]	0.732	1.00	[0.72, 1.37]	0.993

Groups with few subjects were excluded from our sample (*n* = 13). Individuals who were nonbinary or other (*n* = 6), Yellow (*n* = 4) or not declared (*n* = 3) were excluded from the sample, resulting in a sample of 355 participants. The references were the Class A for the income variable and non-Food Addiction group for the Food Addiction variable. Bold type: *p* < .05

Additionally, the magnitude and direction of the association between food addiction severity (based on diagnostic criteria) and UPF consumption remained unchanged when the multivariable model was further adjusted for gender, race, and perceived stress due to the COVID-19 pandemic (Supplementary Table 1).

#### Household income and food addiction symptom presence (based on symptom count)

As shown in Table 3, the bivariate negative binomial model indicated that the presence of food addiction symptoms was significantly associated with higher UPF consumption. Household income was also associated with UPF consumption, with individuals in income classes C and D/E exhibiting higher expected UPF consumption than those in class A. In contrast, no significant difference was observed for class B.

**Table 3:** The impact of income (Classes A, B, C, D/E) and food addiction symptoms (symptomatic or asymptomatic) on UPF consumption: bivariate and multivariable negative binomial regression models

UPF consumption	Bivariate Model			Multivariable Model (Crude)			Multivariable Model (adjusted for BMI)		
	IRR	95% CI	*p* value	IRR	95% CI	*p* value	IRR	95% CI	*p* value
Income									
Class D/E	1.40	[1.09, 1.82]	**0.010**	1.39	[1.08, 1.81]	**0.011**	1.39	[1.08, 1.81]	**0.011**
Class C	1.44	[1.12, 1.87]	**0.005**	1.44	[1.12, 1.86]	**0.005**	1.43	[1.11, 1.85]	**0.006**
Class B	1.09	[0.83, 1.45]	0.526	1.09	[0.83, 1.43]	0.556	1.09	[0.83, 1.44]	0.522
Food addiction									
Symptomatic	1.20	[1.04, 1.37]	**0.010**	1.20	[1.05, 1.37]	**0.008**	1.18	[1.03, 1.35]	**0.018**

Groups with few subjects were excluded from our sample (*n* = 13). Individuals who were Nonbinary or other (*n* = 6), Yellow (*n* = 4) or not declared (*n* = 3) were excluded from the sample, resulting in a sample of 355 participants. The references were the asymptomatic group for the Food Addiction variable or Class A for the income variable. Bold type: *p* < .05

**Fig. 5:**
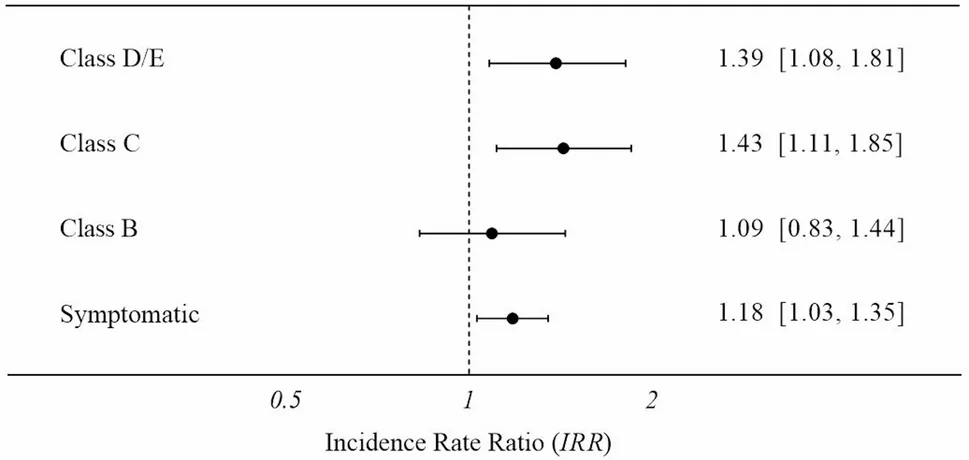
Forest plot of adjusted incidence rate ratios (IRR) for ultra-processed food intake based on food addiction symptom presence and household income (*n* = 355). The Nova-UPF score was the dependent variable. Reference categories were asymptomatic individuals (0 symptoms) and income Class A. Points represent *IRR*s and horizontal lines indicate 95% confidence intervals

In the crude multivariable model including both income and food addiction symptoms, symptom presence remained a significant predictor of UPF consumption, independent of household income. This association persisted after further adjustment for BMI, indicating that participants with one or more food addiction symptoms had approximately 18% higher expected numbers of UPF subgroups consumed the previous day than asymptomatic individuals (*IRR* = 1.18, 95% CI [1.03, 1.35], *p* = .018). As illustrated in Fig. 5, the adjusted incidence rate ratios show that both food addiction symptom presence and lower income classes (C and D/E) were associated with higher UPF consumption, while class B did not differ significantly from the reference group.

Additionally, the magnitude and direction of the association between food addiction symptom presence (based on symptom count) and UPF consumption remained unchanged after further adjustment for gender, race, and perceived stress due to the COVID-19 pandemic (Supplementary Table 2).

### Differences in perceived stress between symptomatic and asymptomatic individuals

**Fig. 6:**
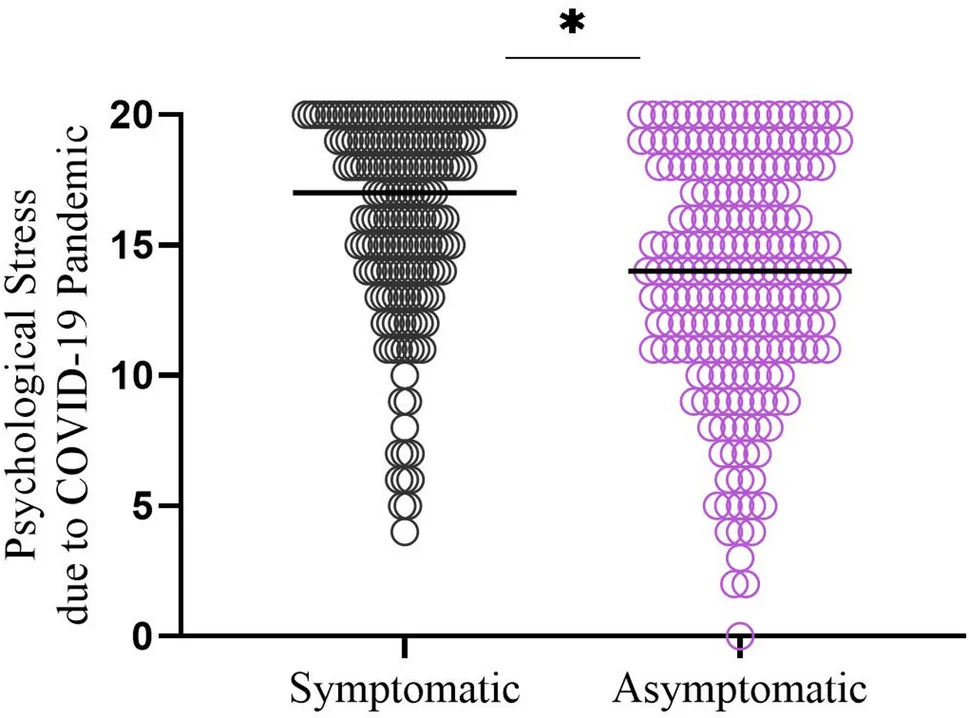
Psychological stress related to the COVID-19 pandemic by food addiction symptom status (*n* = 368). Coronavirus Stress Measure (CSM) scores (range: 0–20) are shown for asymptomatic individuals (0 symptoms) and symptomatic individuals (≥ 1 symptom of food addiction). The horizontal line represents the median; asterisks indicate statistically significant differences (*p* < .050)

Almost half of the population (47.6%) were symptomatic (i.e., presented at least one symptom of food addiction); see Table 1. The Mann‒Whitney *U* test revealed that symptomatic individuals reported greater psychological stress due to the COVID-19 pandemic (as indicated by CSM scale scores) than asymptomatic individuals did (*U* = 11565, *p* < .001; symptomatic *Mdn* = 17, IQR = 5.0; asymptomatic *Mdn* = 14, IQR = 7.0), as shown in Fig. 6.

### Categories of UPF consumed the previous day vs. household income or food addiction symptom presence

A chi-square test showed an association between consumption of products that replace or accompany meals and household income (χ² (3) = 7.84, *p* = .049), with a higher proportion of individuals in Class D/E reporting consumption (84.4%) than in Class A (67.5%). A marginal association was observed between the beverage category and food addiction symptoms (χ² (1) = 3.29, *p* = .070), with higher consumption among symptomatic individuals (65.7%) than asymptomatic individuals (56.5%). No other associations were statistically significant (Tables 4 and 5).

**Table 4:** Nova-UPF score categories (Beverages, products that replace or accompany meals, and snacks) consumed on the previous day according to income classes (A, B, C, D/E).

Nova-UPF Score category	A	B	C	D/E	χ²	*p*
Beverages					4.49	0.210
Yes, *n* (%)	23 (57.5)	44 (54.3)	85 (68.0)	72 (59.0)		
No, *n* (%)	17 (42.5)	37 (45.7)	40 (32.0)	50 (41.0)		
Meals					7.84	**0.049**
Yes, *n* (%)	27 (67.5)	58 (71.6)	91 (72.8)	103 (84.4)		
No, *n* (%)	13 (32.5)	23 (28.4)	34 (27.2)	19 (15.6)		
Snacks					1.97	0.580
Yes, *n* (%)	22 (55.0)	43 (53.1)	78 (62.4)	72 (59.0)		
No, *n* (%)	18 (45.0)	38 (46.9)	47 (37.6)	50 (41.0)		

“Yes” indicates that the participants consumed one or more foods from the subgroups within a category on the previous day. “No” means that the participants did not consume any food from that category on the previous day. Bold type: *p* < .05

**Table 5:** Nova-UPF score categories (beverages, products that replace or accompany meals, and snacks) consumed on the previous day according to food addiction symptoms

Nova-UPF Score category	Symptomatic	Asymptomatic	χ²	*p*
Beverages			3.29	0.070
Yes, *n* (%)	115 (65.7)	109 (56.5)		
No, *n* (%)	60 (34.3)	80 (43.5)		
Meals			0.32	0.570
Yes, *n* (%)	135 (77.1)	144 (74.6)		
No, *n* (%)	40 (22.9)	49 (25.4)		
Snacks			1.49	0.220
Yes, *n* (%)	108 (61.7)	107 (55.4)		
No, *n* (%)	67 (38.3)	86 (44.6)		

“Yes” indicates that the participants consumed one or more foods from the subgroups within a category on the previous day. “No” means that the participants did not consume any food from that category on the previous day

## Discussion

In the present study, we aimed to investigate the impact of food addiction symptoms and household income, both individually (bivariate models) and conjointly (multivariable models), on the consumption of UPF in a Brazilian sample. Overall, we found that both food addiction symptoms and household income were significantly associated with UPF consumption. The presence of one or more food addiction symptoms was associated with greater UPF consumption, as was belonging to income classes D/E or C, compared with class A. These associations remained significant when income and food addiction symptoms were included simultaneously in multivariable models and adjusted for relevant confounders, indicating that each factor contributed independently to UPF consumption.

When food addiction was operationalized using diagnostic criteria, a more nuanced pattern emerged. Only individuals classified as having mild food addiction differed significantly from the non-food addiction reference group, reporting a lower expected number of UPF subgroups consumed on the previous day. In contrast, moderate and severe diagnostic categories were not significantly associated with UPF consumption.

Importantly, differences between symptom-based and diagnosis-based models should be interpreted in light of how food addiction diagnostic criteria are operationalized and how dietary intake was assessed. Diagnostic categories of food addiction (mild, moderate, severe) are defined by threshold-based criteria that combine symptom counts with clinically significant impairment or distress (Schulte & Gearhardt, 2017), resulting in relatively small and behaviorally heterogeneous groups. This heterogeneity is illustrated by the distribution of food addiction severity across BMI categories in our sample (Fig. 2), where mild food addiction was observed across a wide range of BMI levels, including normal weight and overweight individuals, whereas severe food addiction was concentrated in higher obesity classes. In contrast, symptom presence reflects a broader continuum of addictive-like eating tendencies, including subthreshold individuals who may already exhibit heightened sensitivity to the motivational and rewarding properties of UPF. Moreover, UPF intake in the present study was assessed using a 24-hour dietary recall-based measure (Nova-UPF score), which reflects short-term consumption and may be sensitive to day-to-day variability, compensatory restriction, or situational factors (Willett, 2012). Previous research suggests that individuals meeting mild food addiction criteria may engage in intermittent dietary restraint or compensatory control strategies, which may attenuate short-term intake estimates despite the presence of addictive-like symptoms (Gearhardt et al., 2011; Meule & Gearhardt, 2014). Taken together, these factors may explain why symptom presence was positively associated with UPF consumption, whereas the mild diagnostic group showed lower UPF intake on the survey day, without implying inconsistency in the underlying behavioral mechanisms.

In addition, we found that symptomatic individuals reported greater levels of psychological stress related to the COVID-19 pandemic. Given the global mental health burden associated with the pandemic, food addiction symptoms appear to co-occur with broader psychological distress (Cenat et al., 2021; Horsager et al., 2021; Silva-Júnior et al., 2022). Socioeconomic and racial inequalities were also evident: income class D/E was predominantly composed of Black individuals, whereas class A was largely composed of White individuals, reflecting the well-documented racialized structure of income inequality in Brazil (Salata, 2020). Furthermore, a high proportion of individuals in class D/E (84.4%) reported consuming one or more products from the category of foods that replace or accompany meals on the previous day, which may indicate food insecurity and compromised diet quality (Rede Brasileira de Pesquisa em Soberania e Segurança Alimentar e Nutricional, 2022).

Taken together, these findings highlight that UPF consumption in this population is shaped by the intersection of socioeconomic constraints and addictive-like eating behaviors, providing a basis for considering broader public health and policy responses.

### Household income and food addiction symptoms predict the consumption of UPF: implications for policy

Previous research on eating habits and trends over the course of a decade, particularly after the COVID-19 pandemic outbreak, has warned of the increased role that UPF plays in caloric intake in Brazil (Andrade et al., 2023; Louzada et al., 2023; Monteiro et al., 2013). There is robust evidence that the consumption of UPF is positively associated with weight gain and an increased risk of developing noncommunicable diseases (Lane et al., 2024). Recently, it has been estimated in a study conducted by researchers from the University of São Paulo that the number of premature deaths (aged 30 to 69 years) in Brazil associated with the consumption of UPF is approximately 57,000 deaths per year, based on 2019 data (Nilson et al., 2023). Moreover, the study also showed that if the Brazilian population reduced its consumption of UPF by 10%, 20%, or 50%, 5.9 thousand, 12 thousand, or 29.3 thousand lives would be saved per year, respectively (Nilson et al., 2023). Currently, Brazil and other Latin American countries are moving in the opposite direction, with rising UPF consumption (PAHO, 2019).

A determining factor for the increase in UPF intake in Brazil may be the low prices of UPF (Machado et al., 2017), which affects mainly lower-income populations and exacerbates social differences. Our results support this hypothesis since we showed that UPF intake increases by 39% and 42% if individuals belong to Classes D/E and C, respectively, rather than Class A. Thus, differences in the consumption of UPF were found between the lowest and the highest income levels, with the former being composed mainly of black individuals. To increase the value, accessibility, and affordability of freshly prepared meals and unprocessed/minimally processed foods, public health policies and market incentives are necessary (PAHO, 2015). Healthy food should be reasonably priced and consistent in supply. However, Brazilian food environments are becoming increasingly dominated by UPF (Monteiro et al., 2013; PAHO, 2015). Nonetheless, there seems to be an uneven distribution of UPF based on social class and race in different Brazilian food environments; i.e., the availability of UPF is greater in areas with lower income and a larger population of black individuals (Serafim et al., 2022). In fact, disparities in food environments may significantly affect dietary intake in different neighborhoods (Black et al., 2014).

We found that individuals in Class D/E reported greater consumption of meal replacers (e.g., sausages and other processed meats, instant noodles, and ready-to-eat or ready-to-heat meals), which may indicate a reduction in the consumption of unprocessed/minimally processed meats (poultry, eggs, etc.) after the COVID-19 pandemic. For instance, Andrade et al. (2023) demonstrated a shift in the consumption of fresh meat to UPF alternatives, with the loss of purchasing power being a major contributing factor because of the economic crisis brought about by the COVID-19 pandemic. These findings demonstrate the necessity of socioeconomic inequality-aware regulatory measures for food environments. In Brazil, the prevalence of overweight and obesity seems to be inversely correlated with the price of UPF, primarily in the population with the lowest socioeconomic status (Passos et al., 2020). An increase in healthy food intake was also associated with an increase in dietary costs to the lowest income strata in Brazil (Verly et al., 2021). Thus, the public at large, but especially those with low income, should benefit from the promotion of higher taxes on UPF, as well as lower prices and the creation of marketing strategies, which highlight minimally processed or fresh food preparations.

Addressing the growing environmental and health challenges in Brazil requires a fundamental rethinking of food systems (Jaime et al., 2021; Leite et al., 2022). Increasingly, ultra-processed foods are being framed as industrially engineered products analogous to tobacco, designed to maximize reinforcement and habitual consumption, thereby supporting the need for regulatory approaches that extend beyond individual behavior change (Gearhardt et al., 2026). Consumer behavior represents a key leverage point within food systems, as it directly shapes market demand and, consequently, influences what foods are produced and how (Fanzo et al., 2021). Food choices are, in turn, strongly influenced by the positive emotions foods evoke (Gutjar et al., 2015). Ultraprocessed foods are typically high in sugar, salt, and/or fat and are combined with food additives to enhance cosmetic properties and market appeal, often supported by aggressive marketing strategies (Canella et al., 2023). Together, these features create products with heightened emotional appeal, capable of eliciting stronger positive affective motivation than unprocessed or minimally processed foods (David et al., 2018; Lemos et al., 2022) and increased addictive potential (Gearhardt et al., 2023).

Several studies have shown that UPF are associated with addictive-like behaviors in a Brazilian sample (Filgueiras et al., 2019; Silva-Júnior et al., 2023). In the only study conducted in Brazil involving university students, Silva-Júnior et al. (2023) showed that university students with food addiction presented a lower consumption of unprocessed/minimally processed foods and a greater consumption of UPF than did university students without food addiction (Silva-Júnior et al., 2023). The current study is the first to show the impact of food addiction symptom counts on UPF consumption, without accounting for diagnostic criteria. Previous studies have evaluated the relationship between food addiction and UPF intake but considered diagnostic criteria (Schulte & Gearhardt, 2017) since they were interested in the diagnosis of food addiction or different levels of severity of food addiction symptoms (Filgueiras et al., 2019; Silva-Júnior et al., 2023; Whatnall et al., 2022). Given that, by using the food addiction symptoms’ count score, people with one or more addiction symptoms might make up a sizable segment of the population (approximately half of our sample), studies demonstrating how having one or more symptoms already affects UPF consumption are crucial from a public health perspective. For instance, many individuals may be more receptive to the alluring qualities of UPF, such as hyperpalatability and/or advertising (Dagher, 2009), which adds to the debate regarding the Brazilian government’s efforts to regulate the practices of the ultra-processed food UPF industry.

Furthermore, we found that symptomatic individuals reported a marginal but greater consumption of ultra-processed beverages than asymptomatic individuals. Sweetened beverages were described by Moran et al. (2016) as one of the most potentially addictive UPF categories recognized by the general population. They also showed that believing that certain foods are addictive is associated with greater public support for policies aiming to reduce the consumption of those foods (Moran et al., 2016). Thus, the evidence that the consumption of UPF is linked to food addiction symptoms provided herein may help regulatory and policy efforts to reduce addictive patterns of UPF intake.

Since the COVID-19 pandemic, data regarding the relationship between the intake of UPF and food addiction symptoms have gained even more significance. In agreement with the findings of previous studies (e.g., Hong et al., 2020), we found that individuals with one or more food addiction symptoms were more psychologically distressed because of the COVID-19 pandemic than asymptomatic individuals were. Our findings appear to support widespread worries about the detrimental effects of the COVID-19 pandemic on addictive food behaviors, indicating that this issue needs to be monitored and that regulatory measures on the consumption of UPF must be taken.

### Strengths and limitations

The current study has strengths and limitations. The use of the Nova score to assess UPF intake can be considered a strength because it is a validated, replicable, brief, and easy-to-apply method for evaluating population UPF intake in remote studies (Correa-Madrid et al., 2023; Costa et al., 2021). The UPF intake obtained by the Nova-UPF score is equivalent to that obtained with a 24-hour dietary recall administered by a trained nutritionist (Costa et al., 2021) and reflects recent, previous-day intake. However, since the Nova score method is an adaptation of 24-hour dietary recall, it provides limited data about habitual food consumption. In epidemiological studies, food frequency questionnaires are a well-established method of measuring the habitual food consumption of a population. Conversely, food frequency questionnaires and other methods, such as food records, require more time and effort from participants, thereby increasing the complexity of data collection (Marchioni et al., 2019).

Sample size should be considered a limitation of this study. Although the original calculation was based on a prevalence design, the final analytical sample (*n* = 368) was slightly below the number required for a 5% margin of error, which may have limited statistical power for subgroup analyses, particularly those involving moderate and severe food addiction diagnoses.

In addition, by using a sample of undergraduate students from a federal university, we were able to account for sociodemographic variables that affect how the results are interpreted in relation to income. This strategy allowed better internal validity (Andrade, 2018), but caution should be exercised when extrapolating these results to other samples. Although federal public universities in Brazil include students from diverse socioeconomic backgrounds, recruiting from a single institution may limit representativeness. University students differ from other Brazilian youth in educational attainment and access to institutional resources, and the findings may not generalize to young adults not enrolled in higher education. Other studies exploring other sociodemographic variables, such as age, scholarly level, and Brazilian region, are suggested to extend the present findings. It is also important to mention that this was a cross-sectional study, which limited causal inference and was vulnerable to recall bias; as in national surveys conducted by the Brazilian Institute of Geography and Statistics, household income was self-reported and categorized into broad income classes, which may involve some imprecision.

## Conclusion

In conclusion, we observed that the presence of food addiction symptoms and household income contribute conjointly to UPF intake in a sample of Brazilian university students. Moreover, our study corroborated the impact of household income and the presence of food addiction symptoms on the consumption of specific UPF categories, such as products that replace or accompany meals and beverages. Identifying individual groups at risk of excessive UPF consumption may help guide early intervention and public policy measures. Understanding the impact of the presence of food addiction symptoms and household income on UPF intake may provide support for policy approaches in Brazil aimed at reducing the consumption of UPF, such as creating taxes on UPF, reducing unprocessed/minimally processed food prices, and improving the regulation of marketing and front-of-pack nutrition labels.

## Supplementary Information

Supplementary Material 1.

Supplementary Material 2.

Supplementary Material 3.

Supplementary Material 4.

## Data Availability

The current article is accompanied by the relevant raw data generated during and/or analysed during the study. This file is available and accessible as Supplementary Material 4 (raw data).
