# Supplementary material for: Household income and food addiction symptoms predict ultra-processed foods intake in Brazilian university students: implications for policy
Source: Psicol Reflex Crit. 2026 Jul 1;39:32. doi: 10.1186/s41155-026-00405-1 (PMC13593976; doi:10.1186/s41155-026-00405-1)
Supplement: Supplementary file 1 [file 41155_2026_405_MOESM2_ESM.docx]

**Supplemental Table 1**

*The Impact of Income (Classes A, B, C, D/E) and Food Addiction Diagnosis (Mild, Moderate or Severe) on UPF Consumption: Bivariate and Multivariable Negative Binomial Regression Models.*

| **UPF consumption** |  | **Bivariate Model** | | |  | **Multivariable Model (Crude)** | | |  | **Multivariable Model (Adjusted for BMI, gender, race and perceived stress)** | | |
| --- | --- | --- | --- | --- | --- | --- | --- | --- | --- | --- | --- | --- |
|  |  | ***IRR*** | **95% CI** | ***p* value** |  | ***IRR*** | **95% CI** | ***p* value** |  | ***IRR*** | **95% CI** | ***p* value** |
| **Income** |  |  |  |  |  |  |  |  |  |  |  |  |
| Class D/E |  | 1.40 | [1.09, 1.82] | **.010** |  | 1.37 | [1.06, 1.78] | **.016** |  | 1.38 | [1.07, 1.80] | **.016** |
| Class C |  | 1.44 | [1.12, 1.87] | **.005** |  | 1.40 | [1.09, 1.82] | **.010** |  | 1.39 | [1.08, 1.81] | **.012** |
| Class B |  | 1.09 | [0.83, 1.45] | .526 |  | 1.06 | [0.80, 1.40] | .701 |  | 1.05 | [0.80, 1.39] | .715 |
| **Food addiction** |  |  |  |  |  |  |  |  |  |  |  |  |
| Mild  Moderate  Severe |  | 0.71  1.07  1.11 | [0.51, 0.98]  [0.77, 1.48]  [0.80, 1.52] | **.039**  .664  .523 |  | 0.71  1.01  1.06 | [0.51, 0.97]  [0.73, 1.39]  [0.77, 1.44] | **.036**  .932  .732 |  | 0.66  0.99  0.97 | [0.48, 0.92]  [0.71, 1.36]  [0.70, 1.33] | **.014**  .946  .842 |

Note: Groups with few subjects were excluded from our sample (*n* = 13). Individuals who were Nonbinary or other (*n* = 6), Yellow (*n* = 4) or	not declared (*n* = 3) were excluded from the sample, resulting in a sample of 355 participants. The references were the Class A for the	income variable and the non-Food Addiction group for the Food Addiction variable.
