# Supplementary material for: Household income and food addiction symptoms predict ultra-processed foods intake in Brazilian university students: implications for policy
Source: Psicol Reflex Crit. 2026 Jul 1;39:32. doi: 10.1186/s41155-026-00405-1 (PMC13593976; doi:10.1186/s41155-026-00405-1)
Supplement: Supplementary file 2 [file 41155_2026_405_MOESM3_ESM.docx]

**Supplemental Table 2**

*The Impact of Income (Classes A, B, C, D/E) and Food Addiction Symptoms (Symptomatic or Asymptomatic) on UPF Consumption: Bivariate and Multivariable Negative Binomial Regression Models.*

| **UPF consumption** |  | **Bivariate Model** | | |  | **Multivariable Model (Crude)** | | |  | **Multivariable Model (adjusted for BMI, gender, race and perceived stress)** | | |
| --- | --- | --- | --- | --- | --- | --- | --- | --- | --- | --- | --- | --- |
|  |  | ***IRR*** | **95% CI** | ***p* value** |  | ***IRR*** | **95% CI** | ***p* value** |  | ***IRR*** | **95% CI** | ***p* value** |
| **Income** |  |  |  |  |  |  |  |  |  |  |  |  |
| Class D/E |  | 1.40 | [1.09, 1.82] | **.010** |  | 1.39 | [1.08, 1.81] | **.011** |  | 1.42 | [1.09, 1.85] | **.009** |
| Class C |  | 1.44 | [1.12, 1.87] | **.005** |  | 1.44 | [1.12, 1.86] | **.005** |  | 1.44 | [1.12, 1.87] | **.005** |
| Class B |  | 1.09 | [0.83, 1.45] | .526 |  | 1.09 | [0.83, 1.43] | .556 |  | 1.10 | [0.84, 1.45] | .507 |
| **Food Addiction** |  |  |  |  |  |  |  |  |  |  |  |  |
| Symptomatic |  | 1.20 | [1.04, 1.37] | **.010** |  | 1.20 | [1.05, 1.37] | **.008** |  | 1.18 | [1.02, 1.36] | **.023** |

Note: Groups with few subjects were excluded from our sample (*n* = 13). Individuals who were Nonbinary or other (*n* = 6), Yellow (*n* = 4)	or not declared (*n* = 3) were excluded from the sample, resulting in a sample of 355 participants. The references were the	asymptomatic group for the Food Addiction variable or Class A for the income variable.
