# Supplementary material for: Household income and food addiction symptoms predict ultra-processed foods intake in Brazilian university students: implications for policy
Source: Psicol Reflex Crit. 2026 Jul 1;39:32. doi: 10.1186/s41155-026-00405-1 (PMC13593976; doi:10.1186/s41155-026-00405-1)
Supplement: Supplementary file 3 [file 41155_2026_405_MOESM4_ESM.docx]

|  | **Supplemental Table 3** | |  | |  | |  |  |  |
| --- | --- | --- | --- | --- | --- | --- | --- | --- | --- |
|  | *Representativeness of the Research Sample and Total Sample Size at Fluminense Federal University.* | | | | | | |  |  |
|  |  | **Research sample (*n* = 368)** | | **Percentage (%)** | | **Population of the research setting (*N* ≈ 45,000)*** | | **Percentage (%)** |  |
|  | **Total sample size** | 368 | | 100 | | 45.137 | | 100 |  |
|  | **Race** |  | |  | |  | |  |  |
|  | Black | 142 | | 39 | | 16.091 | | 36 |  |
|  | White | 219 | | 60 | | 20.484 | | 45 |  |
|  | Yellow | 3 | | 1 | | 253 | | 1 |  |
|  | Nondeclared | 4 | | 1 | | 8.260 | | 18 |  |
|  | Indigenous | 0 | | 0 | | 49 | | 0 |  |
|  | **Gender** |  | |  | |  | |  |  |
|  | Male | 115 | | 31 | | 21.434 | | 47 |  |
|  | Female | 247 | | 67 | | 23.703 | | 53 |  |
|  | Nonbinary or other | 6 | | 2 | | 0** | | - |  |
|  | **Household Income** |  | |  | |  | |  |  |
|  | Class D/E | 122 | | 33 | | 11.107 | | 25 |  |
|  | Classes A/B/C | 246 | | 67 | | 27.179 | | 60 |  |
|  | Not applicable | 0 | | 0 | | 6.851 | | 15 |  |
|  | * The total sample size has fluctuated between data collection (*N* ≈ 40.000) and the table's completion (*N* ≈ 45.000).   ** The censuse only included binary response categories “Female” and “Male”   Source: https://www.uff.br/proplan/uff-em-numeros/ | | | | | | | |  |
